# Recent Advances in Lung Cancer Immunotherapy: Input of T-Cell Epitopes Associated With Impaired Peptide Processing

**DOI:** 10.3389/fimmu.2019.01505

**Published:** 2019-07-03

**Authors:** Marine Leclerc, Laura Mezquita, Guillaume Guillebot De Nerville, Isabelle Tihy, Ines Malenica, Salem Chouaib, Fathia Mami-Chouaib

**Affiliations:** ^1^INSERM UMR 1186, Integrative Tumor Immunology and Genetic Oncology, Gustave Roussy, EPHE, PSL, Faculté de Médecine - Université Paris-Sud, Université Paris-Saclay, Villejuif, France; ^2^Department of Medical Oncology, Gustave Roussy Cancer Campus, Institut d'Oncologie Thoracique, Gustave Roussy, Université Paris-Saclay, Villejuif, France

**Keywords:** lung cancer, cancer immunotherapy, cancer vaccine, tumor escape, antigen presentation and processing, T-cell epitope associated with impaired peptide processing

## Abstract

Recent advances in lung cancer treatment are emerging from new immunotherapies that target T-cell inhibitory receptors, such as programmed cell death-1 (PD-1). However, responses to anti-PD-1 antibodies as single agents are observed in fewer than 20% of non-small-cell lung cancer (NSCLC) patients, and immune mechanisms involved in the response to these therapeutic interventions remain poorly elucidated. Accumulating evidence indicates that effective anti-tumor immunity is associated with the presence of T cells directed toward cancer neoepitopes, a class of major histocompatibility complex (MHC)-bound peptides that arise from tumor-specific mutations. Nevertheless, tumors frequently use multiple pathways to escape T-cell recognition and destruction. In this regard, primary and acquired resistance to immune checkpoint blockade (ICB) therapy was associated with alterations in genes relevant to antigen presentation by MHC-class I/beta-2-microglobulin (MHC-I/β2m) complexes to CD8 T lymphocytes. Among additional known mechanisms involved in tumor resistance to CD8 T-cell immunity, alterations in transporter associated with antigen processing (TAP) play a major role by inducing a sharp decrease in surface expression of MHC-I/β2m-peptide complexes, enabling malignant cells to evade cytotoxic T lymphocyte (CTL)-mediated killing. Therefore, development of novel immunotherapies based on tumor neoantigens, that are selectively presented by cancer cells carrying defects in antigen processing and presentation, and that are capable of inducing destruction of such transformed cells, is a major challenge in translational research for application in treatment of lung cancer. In this context, we previously identified a non-mutant tumor neoepitope, ppCT_16−25_, derived from the preprocalcitonin (ppCT) leader sequence and processed independently of proteasomes/TAP by a mechanism involving signal peptidase (SP) and signal peptide peptidase (SPP). We also provided *in vitro* and *in vivo* proof of the concept of active immunotherapy based on ppCT-derived peptides capable of controlling growth of immune-escaped tumors expressing low levels of MHC-I molecules. Thus, non-mutant and mutant neoepitopes are promising T-cell targets for therapeutic cancer vaccines in combination with ICB. In this review, we summarize current treatments for lung cancer and discuss the promises that conserved neoantigens offer for more effective immunotherapies targeting immune-escaped tumor variants.

## Introduction

Lung cancer is the third most frequent cancer worldwide and the leading cause of cancer-related death in Europe and the United States because of its frequency and resistance to currently available treatments (1). About 80–85% of human lung cancers belong to the category of non-small cell lung cancer (NSCLC), including squamous cell carcinoma (SCC), adenocarcinoma (ADC) and large-cell carcinoma (LCC). The remaining 15–20% include small cell lung cancer (SCLC), the most aggressive subtype of lung cancer. In the NSCLC setting, surgery resection is the classical treatment in early stages; chemotherapy in combination with radiotherapy is proposed in locally advanced cancer, and chemotherapy has been the standard of care in advanced diseases over the last 30 years. However, under this management, fewer than 18% of patients reach 5 years of expected survival (2). Particularly in advanced disease, novel therapeutic strategies such as targeted therapies against oncogenic driver alterations or immunotherapy agents have changed the paradigm of NSCLC patients, and are currently being evaluated in combination with other treatments at all stages so as to establish the best standard of care for these cancer patients. However, tumors frequently develop resistance mechanisms to these promising new therapies. Among known mechanisms involved in tumor escape from T-cell-based immunotherapies, alterations in antigen presentation by major histocompatibility complex (MHC) molecules play an important role. In this context, therapeutic cancer vaccines based on T-cell epitopes generated independently of transporter associated with antigen processing (TAP), such as leader sequence-derived peptides, correspond to promising strategies to eradicate tumors with impaired antigen processing and presenting machinery (APM), and thus to overcome tumor escape from CD8 T-cell immunity.

## Conventional Chemotherapy and Targeted Therapy in Lung Cancer

Platinum-based doublet chemotherapy is the standard first-line treatment for non-selected patients with advanced NSCLC who have a good performance status (3). Patients are treated either with cisplatin or carboplatin, where four to six cycles are administered independently of NSCLC histology (4). Although a benefit in time to progression was observed, this did not translate into a significant decrease in mortality. In preclinical models, it was shown that these chemotherapeutic agents can potentiate immune responses toward cancer cells by increasing the cancer cell mutational load (5) and enhancing the human leukocyte antigen (HLA) expression level (6), leading to better recognition of cancer cells by cytotoxic T lymphocytes (CTL). Moreover, reduced activity of myeloid-derived suppressor cells (7) and enhanced expression of PD-L1 on cancer cells (8) were described following neo-adjuvant chemotherapeutic treatments in patients with SCC. These data may reinforce strategies aimed at combining chemotherapy with immunotherapy in lung cancer.

Approximately 20% of NSCLC tumors have a druggable oncogenic driver, such as the epidermal growth factor receptor (*EGFR*) mutation and anaplastic lymphoma kinase (*ALK*) rearrangement, reported in 11 and 5% of NSCLC, respectively (9). Therapies with EGFR and ALK tyrosine kinase inhibitors (TKI) are standard-first line treatments in *EGFR*-mutant and *ALK*-rearranged NSCLC patients, respectively (10, 11). TKI are non-peptide compounds displaying homologies with the adenosine triphosphate (ATP), permitting competition for the ATP-binding domain of protein kinases, thereby preventing phosphorylation and activation of intracellular signaling pathways, resulting in inhibition of proliferation and apoptosis of cancer cells (12, 13). Results from different phase III clinical trials have reported that these personalized treatments improve the response rate, progression-free survival and quality of life compared to standard first-line platinum-based chemotherapy (10, 14). Improved outcome has also been reported with a combination of antiangiogenic agents plus chemotherapy over chemotherapy as second-line treatment in NSCLC patients, most likely by enhancing cancer cell death (15, 16). However, the efficacy of these drugs is limited, with a response rate of <10%.

## Lung Cancer Immune Checkpoint Blockade Immunotherapies

Recent advances in cancer treatment are emerging from new therapies that target T-cell inhibitory receptors, such as cytotoxic T-lymphocyte-associated antigen-4 (CTLA-4) and programmed cell death-1 (PD-1) (17, 18). Indeed, lung cancer immunotherapy has greatly benefitted from the latest mechanistic understanding of inhibitory molecules expressed on the T-lymphocyte surface and involved in modulating antigen-specific T-cell responses. This led to development of promising novel immunotherapies, especially blocking anti-CTLA-4 and anti-PD-1 monoclonal antibodies (mAb), which inhibit interaction between the inhibitory receptor on T cells and its ligands on cancer cells, thereby reestablishing T-cell reactivity and anti-tumor effector functions. These immunotherapies have reported an overall survival benefit compared to standard second-line chemotherapy (docetaxel), and recently, also, in first line platinum-based chemotherapy. During early clinical trials in NSCLC patients treated with anti-PD-1 or anti-PD-1 ligand (PD-L)1 mAb, response rates ranged from 16 to 23%, and some patients showed prolonged disease stability with unprecedented long-term survival (19–21). For instance, the anti-PD-1 nivolumab, as second-line therapy in advanced NSCLC compared with docetaxel, was reported to improve the response rate and overall survival in two randomized phase III trials (22, 23). Pembrolizumab, another anti-PD-1, prolongs survival compared with docetaxel as second-line treatment, with at least 1% of tumor cells expressing PD-L1 (24); and as a front line, pembrolizumab has demonstrated improved survival and response compared to platinum-based chemotherapy if ≥50% of tumor cells express PD-L1 (21). Since NSCLC is generally considered resistant to immunotherapy, these results are rather promising. Another attractive feature of these therapies is that both anti-PD-1 and anti-PD-L1 mAb are well-tolerated, with little grade 3–4 toxicity. Moreover, the success of the immune checkpoint blockade (ICB) appears to extend to other thoracic tumors such as SCLC, mesothelioma and epithelial thymic tumors (25).

A combination of different immune checkpoint inhibitors, such as anti-CTLA-4 plus anti-PD-1, has showed a manageable tolerability profile with anti-tumor activity and clinical benefits (26). Preliminary trials assessing combination therapies with anti-PD-1/PD-L1 plus anti-CTLA-4 have documented considerable advantages in survival indices over single-agent immunotherapy (27, 28). In particular, in lung cancer with a high tumor mutational burden (TMB), defined as ≥10 mutations/megabase (Mb) and used as a novel predictive biomarker of response to immunotherapy (29), a combination of nivolumab plus ipilimumab demonstrated better outcome than chemotherapy in first-line NSCLC patients (30, 31). As CD8^+^ tumor-infiltrating T lymphocytes (TIL) express a large panel of immune checkpoints, including TIM-3, LAG-3, TIGIT, and BTLA (32, 33), several neutralizing antibodies toward these inhibitory receptors are under development for use as drugs in patients with cancer, including lung cancer. Moreover, since each of these immune checkpoints uses a particular mechanism to negatively regulate CD8 T-cell function, studies are attempting to combine different neutralizing antibodies to potentiate the response to immunotherapy. For instance, a combination of anti-PD-1 plus anti-LAG-3 is currently in phase II clinical trials in patients with several solid cancer types, including NSCLC (NCT03365791), as well anti-PD-1 plus anti-TIM-3 antibodies (NCT03708328) and anti-PD-1 plus anti-TIGIT (NCT03563716).

However, independently of the ICB subtype (24, 34, 35), the overall response rate in second-line treatment for unselected NSCLC patient populations is <20%. In addition, predictive response factors, such as PD-L1 expression ≥50% tested by immunohistochemistry, and immune mechanisms involved in the response to these therapeutic interventions, remain elusive and poorly elucidated. Accumulating evidence indicates that effective anti-tumor immunity is associated with the presence of T cells directed toward cancer neoepitopes, a class of MHC-bound peptides that arise from tumor-specific mutations (36–38). These neoantigens are highly immunogenic, since they are not expressed by normal tissues and thus bypass central thymic tolerance. Unfortunately, spontaneous T-cell responses to these mutation-derived tumor antigens are inefficient. Indeed, tumors frequently use multiple pathways to escape immune recognition and elimination. In this regard, primary and acquired resistance to ICB therapies was associated with alterations in genes relevant to antigen presentation by MHC class I/beta-2-microglobulin (MHC-I/β2m) complexes to CD8^+^ T cells (39–41). Mutations in the Janus kinase (JAK)1/JAK2 and interferon (IFN) signaling pathway resulted in the inability to respond to IFN-γ, and thus, the impossibility of improving antigen presentation by MHC-I molecules (39, 42, 43). Moreover, clonal deletion of tumor-specific T cells by IFN-γ was recently reported to confer resistance to ICB therapies (44). Therefore, major efforts are currently being made to develop active immunotherapies to stimulate tumor-specific T cells and induce their proliferation, so as to eliminate malignant cells, including resistant tumor variants.

## Tumor-Associated Antigen-Based and Mutant Antigen-Based Therapeutic Cancer Vaccines

CTL are the main effectors of the immune system, capable of destroying transformed cells following recognition, by the T-cell receptor (TCR), of specific epitopes presented by MHC-I/β2m complexes. Thus, several active immunotherapies have been generated to trigger strong and persistent anti-tumor CTL responses in order to destroy primary tumors and metastases. Current cancer vaccines consist of activating tumor-specific T cells via therapeutic vaccination of cancer patients with tumor-associated antigens (TAA), such as cancer testis antigens and differentiation antigens (45, 46), mutant antigens (47, 48), and viral antigens (49, 50). TAA are relatively restricted to cancer cells, and, to a limited degree, to normal tissues; whereas mutant antigens, also classified as tumor-specific antigens (TSA) or neoantigens, are expressed only in cancer cells, arising from gene mutations that result in novel abnormal protein synthesis (51). Optimal therapeutic cancer vaccines include synthetic long peptides derived from these antigenic proteins administered with an adjuvant, as well as DNA and RNA vaccines delivered to both MHC-I and MHC-II molecules of professional antigen-presenting cells (APC), namely dendritic cells (DC), thereby promoting both CD8 and CD4 T-cell responses in patients with cancers (50, 52, 53).

Thus far, therapeutic vaccination with TAA, such as MAGE-A3, PRAME, or MUC1, had not revealed objective cancer regression, nor an increase in disease-free survival in patients with NSCLC (54–57). However, improved survival in NSCLC was observed in some patients and correlated with development of CD8 T-cell responses against targeted TAA and non-targeted TAA, demonstrating induction of epitope spreading (58). The era of more successful therapeutic vaccines has arrived, with accessibility to next-generation sequencing (NGS) technology and *in silico* epitope prediction, contributing to identification of patient-specific tumor antigens generated by somatic mutations in individual tumors. These neoantigens have opened up new perspectives in therapeutic cancer vaccines against a wide variety of cancer types, and possibly also against virally induced epithelial cancer (59). Vaccination directed against these tumor-encoded amino acid substitutions resulted in an increase in antigenic breadth and clonal diversity of neoantigen-specific T lymphocytes in melanoma (60). Moreover, clinical benefits of neoantigen-based cancer vaccines have been observed in melanoma patients (47, 48). This neoantigen-based strategy is now in development in many cancers, including lung cancer, with tumor regression observed in a SCC patient (61). A feasibility study of personalized neoepitope peptide vaccination has also been performed on advanced SCLC patients, with possible prolongation of overall survival (62). In another randomized phase II study, personalized peptide vaccines in combination with docetaxel did not improve survival of patients with previously treated advanced wild-type EGFR NSCLC (63). This may be due to the multiple resistance pathways developed by the tumor itself to evade the induced neoantigen-specific T-cell response. In this regard, acquired resistance to a neoepitope-based cancer vaccine associated with the outgrowth of β2m-deficient cancer cells was observed in a vaccinated melanoma patient (47).

## Promising Non-mutant Neoepitope-Based Cancer Vaccines

Most antigenic peptides derived from TAA and recognized by CD8 T lymphocytes originate from cleavage in proteasomes of intracellular proteins and their transport, by TAP, from the cytosol into the endoplasmic reticulum (ER) (64). The resulting 9-to-10-amino acid peptides bind to MHC-I molecules and are then presented on the surface of transformed cells for CD8 T-cell recognition. Among known mechanisms frequently used by cancer cells to evade CTL recognition and killing, alterations in TAP play a major role by inducing a sharp decrease in surface expression of MHC-I/β2m-peptide complexes, enabling malignant cells to become “invisible” to CD8 T cells (65–71). Downregulation of MHC-I molecules has been found to range from 16 to 50% among primary lesions from various types of human carcinomas (72). Moreover, between 39 and 88% of human tumors were reported to be MHC-I deficient, including 73% of lung cancers with a total loss of class I molecules in 38% and loss of A locus and A2 allele in 8.3 and 27% of the analyzed cases, respectively (73, 74); for a review see (75). Our earlier studies have stressed downregulation of TAP1 and/or TAP2 in lung cancer cells, resulting in resistance to TCR-dependent lysis (76). TAP deficiencies have been observed in a wide variety of human cancers, including cervical carcinoma (68), head and neck carcinoma (69), melanoma, gastric cancer (65, 66), and lung cancer, with up to 70% of NSCLC expressing low levels of TAP1 and/or TAP2 (77), and are associated with tumor escape from immune system control. Therefore, the discovery of alternative pathways for tumor antigen processing may improve anti-tumor T-cell responses and T-cell-based immunotherapy strategies. In this regard, the generation of tumor-specific CTL epitopes from PRAME and melanoma antigen MART-1 required cytosolic endopeptidases nardilysin and thimet oligopeptidase (78). A CD8 T-cell epitope derived from MAGE-A3 TAA processed independently of the proteasome by the insulin-degrading enzyme has been described (79). Moreover, a CTL epitope derived from the signal sequence of melanoma-associated tyrosinase is processed by a proteasome/TAP-independent pathway (80). These tumor epitopes appear to be promising targets for active immunotherapy to induce CTL responses toward APM-impaired tumors.

Remarkably, T Van Hall's group previously reported that T lymphocytes specific to a murine non-mutated self-epitope, derived from the C-terminus region of the TRH4 protein and defined as a T-cell epitope associated with impaired peptide processing (TEIPP), were selected in the thymus of TCR-transgenic mice and activated by peptide-based vaccination, leading to progression control of TAP-deficient tumors expressing low levels of MHC-I/peptide complexes (81). Using a combinatorial screening approach, this group more recently identified human non-mutated neoantigens presented by TAP-deficient cancer cells (82). TEIPP neoantigens are derived from ubiquitous non-mutant self-proteins that are not naturally loaded into MHC-I molecules of healthy cells, because they express standard levels of TAP. Their surface presentation is induced in transformed cells following downregulation or loss of TAP (82). Thus, targeting these non-mutated neoantigens is a promising potent approach to inducing specific responses to tumors displaying low MHC-I expression associated with downregulation or loss of TAP subunits.

**Figure 1:**
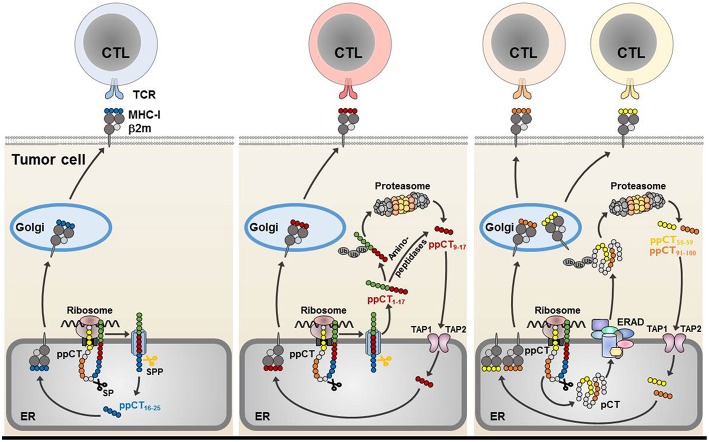
Processing of CD8 ppCT T-cell epitopes. The ppCT signal peptide has a type II orientation (the NH2-terminal region exposed toward the cytosol and the COOH-terminal region facing the endoplasmic reticulum lumen). The ppCT_16−25_ epitope is processed by SP and SPP independently of TAP, and is released directly into the endoplasmic reticulum **(left)**. After cleavage by SP and SPP, a ppCT_1−17_ signal peptide fragment is released into the cytoplasm, to be processed by the proteasome/TAP pathway so as to give rise to the ppCT_9−17_ epitope. The ppCT_9−17_ epitope may also be generated from a ppCT_1−17_ signal peptide fragment by cytosolic proteases before transport into the endoplasmic reticulum lumen by TAP **(middle)**. ppCT_50−59_ and ppCT_91−100_ epitopes are generated by the TAP/proteasome pathway after retrotranslocation of the pCT precursor protein from the endoplasmic reticulum lumen into the cytosol by the ERAD pathway **(right)**. All generated ppCT epitopes bind within the endoplasmic reticulum to HLA-A2 molecules before externalization to the target cell membrane for recognition and elimination by specific CD8 T lymphocytes. ER, endoplasmic reticulum; MHC-I, MHC-class I; Ub, ubiquitin.

In humans, we had previously identified a non-mutated tumor neoepitope recognized on a NSCLC tumor cell line by autologous CTL clones isolated from TIL of a lung cancer patient. This epitope (ppCT_16−25_) is generated from the carboxy-terminal region of the preprocalcitonin (ppCT) signal sequence, and is processed by a mechanism independent of proteasomes and TAP, involving signal peptidase (SP) and signal peptide peptidase (SPP) (76, 83). It is released in the ER lumen and presented to CTL by MHC-I molecules on the surface of immune-escaped cancer cells expressing low levels of HLA-class I due to defects in TAP expression. We showed that most human lung tumors frequently express ppCT self-antigen and display altered expression of TAP molecules. Using epitope prediction software, we also showed that ppCT includes additional HLA-A2-restricted T-cell epitopes that are processed by TAP-dependent pathways. Processing occurs in the cytosol, either after retrotranslocation of a procalcitonin (pCT) substrate by the ER-associated degradation (ERAD) pathway (ppCT_50−59_ and ppCT_91−100_) or release of a signal peptide precursor by SPP (ppCT_9−17_) (77). Thus, CD8 T-cell epitopes from signal peptides with a type II signal anchor (84), like the ppCT leader sequence (83, 85), are released into the ER lumen and presented to CTL by MHC-I in a TAP-independent manner (such as the ppCT_16−25_), or into the cytoplasm where they are further processed by the proteasome or cytosolic proteases (like the ppCT_9−17_). The latter epitope is then transported by TAP into the ER, where it binds to the HLA-A2 molecules and is then conveyed to the cell surface to be recognized by CD8 T cells (Figure 1).

To resolve the tumor heterogeneity question regarding the TAP expression level, we selected a cocktail of five ppCT immunogenic peptides, including the HLA-A2-restricted TAP-independent ppCT_16−25_ neoepitope, the two ppCT epitopes processed by TAP-dependent pathways (ppCT_9−17_ and ppCT_50−59_) and two ppCT long peptides (ppCT_1−15_ and ppCT_86−100_). We provided *in vivo* proof of concept of a therapeutic cancer vaccine based on this ppCT peptide cocktail, delivered with poly(I:C) adjuvant, which was capable of inducing anti-tumor CTL responses in HLA-A^*^0201/HLA-DR3-transgenic (HHD-DR3) mice and in NOD-*scid-Il2r*γ^*null*^ (NSG) mice adoptively transferred with human HLA-A2^+^ peripheral blood mononuclear cells (PBMC), resulting in growth control of established lung tumors expressing low levels of HLA-A2/ppCT peptide complexes (77). Thus, non-mutant TEIPP, such as ppCT_16−25_, represent attractive candidates for more effective cancer immunotherapies and combination therapies aimed at APM-impaired tumor variants. Signal sequence-derived peptides correspond to promising targets for therapeutic cancer vaccines against TAP-deficient tumor cells. Indeed, these TAP-independent self-peptides are not presented by normal cells displaying a standard processing status and emerge at the surface of tumor cells following alterations in APM components. This might explain their immunogenicity and ability to trigger effective anti-tumor T-cell responses.

## Concluding Remarks

Personalized RNA mutanome vaccines and multipeptide neoantigen vaccines have recently demonstrated their efficacy in melanoma (47, 48). Active immunotherapies with predicted tumor neoepitopes were even more efficient when combined with anti-PD-1 mAb. However, recurrence associated with defects in APM was frequently observed. In this context, TEIPP-specific T cells are valuable effectors against immune-edited MHC-I^low^ targets that have acquired resistance to immunotherapy (86). Signal-sequence-derived peptides and their carrier proteins, such as ppCT, are attractive candidates for specific therapeutic cancer vaccines to target tumors that had downregulated HLA-class I molecules due to alterations in TAP expression. Cancer vaccines based on non-mutant neoantigens presented by TAP-deficient tumors expressing low levels of MHC-I/peptide complexes, in association with individualized tumor mutanome epitopes, represent a major challenge in lung cancer. The specificity of therapeutic vaccination with mutant and non-mutant neoepitopes combined with ICB offers an attractive strategy for future cancer immunotherapies. A combination of such active immunotherapies with ICB would permit expansion of pre-existing antigen-specific T cells and induction of a broader repertoire of T-cell specificities, enhancing tumor progression control and leading to elimination of most cancer cells, including immune-escaped variants, and destruction of ≪ immune deserts or cold tumors ≫ that are weakly infiltrated by lymphocytes. ICB, such as anti-PD-1 and anti-PD-L1 mAb, would optimize the anti-tumor activities of cancer-vaccine-induced T cells by inhibiting the interaction of PD-1 on these activated tumor-specific T cells with PD-L1/-L2 on the target cell surface, overcoming T-cell exhaustion and resulting in malignant cell eradication.

## Author Contributions

ML and FM-C coordinated the writing of the manuscript and participated in drafting and editing the text and figure. All authors gave final approval to the version submitted. LM, GG, IT, IM, and SC participated in writing the manuscript.

### Conflict of Interest Statement

The authors declare that the research was conducted in the absence of any commercial or financial relationships that could be construed as a potential conflict of interest.

## Abbreviations

APM: antigen presentation and processing machinery
β2m: beta-2-microglobulin
CTL: cytotoxic T lymphocyte
CTLA: cytotoxic T-lymphocyte-associated antigen
ER: endoplasmic reticulum
HLA: human leukocyte antigen
IFN: interferon
ICB: immune checkpoint blockade
mAb: monoclonal antibody
MHC-I: major histocompatibility complex-class I
NSCLC: non-small-cell lung carcinoma
PD-1: programmed cell death-1
ppCT: preprocalcitonin
TEIPP: T-cell epitope associated with impaired peptide processing
TIL: tumor-infiltrating T lymphocyte
TCR: T-cell receptor.
